# Dopaminergic and Serotonergic Drug Use: A Nationwide Register-Based Study of Over 1 300 000 Older People

**DOI:** 10.1371/journal.pone.0023750

**Published:** 2011-08-15

**Authors:** Kristina Johnell, Håkan Fischer

**Affiliations:** 1 Aging Research Center, Karolinska Institutet and Stockholm University, Stockholm, Sweden; 2 Department of Psychology, Stockholm University, Stockholm, Sweden; Innsbruck Medical University, Austria

## Abstract

**Objective:**

To investigate the use of dopaminergic and serotonergic drugs in elderly people.

**Methods:**

We analyzed data on age, sex and dispensed drugs for individuals aged ≥65 years registered in the Swedish Prescribed Drug Register from July to September 2008 (n = 1 347 564; 81% of the total population aged ≥65 years in Sweden). Main outcome measures were dopaminergic (enhancing and/or lowering) and serotonergic (enhancing and/or lowering) drugs and combinations of these.

**Results:**

Dopaminergic and serotonergic drugs were used by 5.6% and 13.2% the participants, respectively. Female gender was related to use of both dopaminergic and, particularly, serotonergic drugs. Higher age was associated with use of dopamine lowering drugs and serotonergic drugs, whereas the association with use of dopamine enhancing drugs declined in the oldest old. The occurrence of combinations of dopaminergic and serotonergic drugs was generally low, with dopamine lowering + serotonin lowering drug the most common combination (1.6%). Female gender was associated with all of the combinations of dopaminergic and serotonergic drugs, whereas age showed a mixed pattern.

**Conclusion:**

Approximately one out of ten older patients uses serotonergic drugs and one out of twenty dopaminergic drugs. The frequent use of dopaminergic and serotonergic drugs in the elderly patients is a potential problem due to the fact that aging is associated with a down-regulation of both these monoaminergic systems. Future studies are needed for evaluation of the impact of these drugs on different cognitive and emotional functions in old age.

## Introduction

The use of pharmaceutical drugs in today's society is unevenly distributed across different age cohorts. Over 90% of older people (≥70 years) use drugs compared to less than 65% in younger age groups (<50 years) [1]. Many drugs affect one or more of the monoaminergic transmitter systems that have important roles in different cognitive and emotional processes. This could, in turn, result in hampered function in these cognitive and affective domains, as side effects of the drug [2]. Given that age-related changes in cognition and emotion are in part a consequence of age-related changes in the brain, it is of interest to investigate how frequent the use of drugs is that alter dopaminergic and serotonergic function in older adults brains.

Two monoaminergic systems that have important roles in cognitive and affective processing are the dopaminergic and the serotonergic transmitter systems. Many studies have shown that dopamine is strongly involved in higher cognitive function [3], [4] and that aging is associated with a loss of striatal and extrastriatal biomarkers [5], [6]. Some studies have even indicated a curvelinear trajectory with accelerated dopamine losses from young-old to very old age [7]–[9] and that these age-related dopamine losses are related to age-related deficits in several different cognitive abilities [10]. Experimental data now support this latter claim [11]. Studies in humans have also shown significant decline in the serotonergic system in normal aging, important for both emotional function [12], [13] and dyfunction [14], [15], that are independent of disease state [16], [17]. Recent research also implicate a role for serotonin in the NMDA receptor antagonist models of cognitive impairment [18]. The importance of balance between the dopaminergic and the serotonergic systems for working memory function gives further evidence for involvement of the serotonin system also in cognitive function [19].

Little is known about the extent of use of dopaminergic and serotonergic drugs in the general elderly population. However, the prevalence of use of certain types of dopaminergic and serotonergic drugs has been estimated. For instance, recent studies have reported prevalences of 12–20% for antidepressant [20]–[23], 5–12% for antipsychotic [20], [21], [24] and 2% for L-dopa [21] use in older people.

The aim of the present study was to investigate dopaminergic and serotonergic drug use in a very large population of elderly people in a typical modern industrial society (Sweden). These data will demonstrate how many elderly persons who are potentially cognitively and emotionally affected by drugs that either enhance or decrease function in the dopaminergic or serotonergic system.

## Methods

### Ethics Statement

This study was approved by the ethical board at the Karolinska Institutet in Stockholm (dnr 2009/477-31/3) and we only analyzed de-identified data.

### Study population

The Swedish Prescribed Drug Register contains individual-based data for all prescriptions dispensed to the whole population of Sweden (about 9 million inhabitants) [25]. We analyzed non-identifiable data from individuals aged ≥65 years who were registered in the Swedish Prescribed Drug Register from July to September 2008 (n = 1 347 564), with information on every individual's age, sex and dispensed drugs (amount of prescribed drug, when the prescription was filled, and prescribed dosage according to the prescriptions written by the prescribers).

Information from the 3 month period about when the prescription was filled, the amount of drug and the prescribed dosage was processed to calculate the duration of drug exposure [26]–[28]. The time period of 3 months was chosen based on the fact that drugs are prescribed for use for at most 90 days in Sweden. When prescribed dosage was incomplete or missing, we based our calculations of drug exposure on defined daily doses (DDDs) [29]. For each drug, the mean prescribed daily dose (PDD) [29] for regular use was calculated. In the few cases where the PDD could not be calculated, we assumed 0.9 DDDs for regularly used drugs (based on calculations of the total mean value for regularly used drugs among the elderly in the study population). For drugs prescribed as needed, 0.45 DDDs (50% of 0.9) was employed. For dermatological and eye preparations, 1 DDD was assumed [27], [28]. A list of current prescriptions was constructed based on the calculations of the duration of drug exposures for every individual on the arbitrarily chosen date 30 September 2008. If a patient was dispensed the same drug in different doses during the study period, this was counted as one dispensed drug.

### Definitions

Drugs were classified according to the Anatomical Therapeutic Chemical (ATC) classification system [29], as recommended by the World Health Organization. The classification of dopaminergic (i.e. enhancing and/or lowering) and serotonergic (i.e. enhancing and/or lowering) drugs [30], [31] is shown in Table 1.

**Table 1:** Classification of dopaminergic and serotonergic drugs.

ATC-class	Drug type
Dopaminergic drugs	
Dopamine enhancing drugs	
N04B	Anti-Parkinson dopaminergic drugs
N06AF	Monoamine oxidase inhibitors, non-selective
N06AG	Monoamine oxidase A inhibitors
N06AX16	Venlafaxine
N06AX21	Duloxetine
N06B	Psychostimulants, agents used for ADHD and nootropics
Dopamine lowering drugs	
N05A	Antipsychotics
N05BE01	Buspirone
A03FA01	Metoclopramide
Serotonergic drugs	
Serotonin enhancing drugs	
N06A	Antidepressants
N02CC	Antimigraine selective serotonin (5HT1) agonists
Serotonin lowering drugs	
N05AE	Atypical antipsychotics; Indole derivatives
N05AH	Atypical antipsychotics; Diazepines, oxazepines, thiazepines and oxepines
N05AX	Atypical antipsychotics; Other antipsychotics

Age was categorized into six groups: 65–69, 70–74, 75–79, 80–84, 85–89 and ≥90 years.

### Statistical analysis

We analyzed the occurrence of dopaminergic and serotonergic drug use in relation to age and sex. We also investigated the occurrence of the following combinations of dopaminergic and serotonergic drugs: dopamine enhancing + dopamine lowering drug, serotonin enhancing + serotonin lowering drug, dopamine enhancing + serotonin enhancing drug and dopamine lowering + serotonin lowering drug.

Logistic regression analysis was used to analyze whether age and sex were associated with use of dopaminergic and serotonergic drugs and combinations of these. The results are expressed as odds ratios (ORs) with 95% confidence intervals (CIs). PASW Statistics 18 for Windows was used for the analyses.

## Results

In the study population, mean age was 76.2 years (range 65–109 years), 58% were women and 4.5 drugs were on average used per person (Table 2).

**Table 2:** Characteristics of the 1 347 564 older people, 2008.

Mean age, years ± SD	76.2±7.9
Sex, % (n)	
Women	57.5 (775 109)
Men	42.5 (572 455)
Mean number of dispensed drugs, no. ± SD	4.5±3.3

Dopaminergic drugs were used by 5.6% of the participants and they were most commonly used by the oldest old (Table 3). The most frequently used dopaminergic drugs were antipsychotics (2.8%) and anti-Parkinson dopaminergic drugs (1.8%). Use of dopamine enhancing drugs were about as common as use of dopamine lowering drugs. Use of ≥2 dopamine enhancing (0.6%) or ≥2 dopamine lowering drugs (0.3%) was uncommon. Serotonergic drugs were used by 13.2% of the elderly. The most frequently used serotonergic drugs were antidepressants (12.3%) and atypical antipsychotics (1.2%). Use of serotonergic drugs increased with age and 23.0% of people aged ≥90 years used these drugs. Serotonin enhancing drugs were more commonly used than serotonin lowering drugs, particularly in women. Use of ≥2 serotonin enhancing (1.7%) or ≥2 serotonin lowering drugs (0%) was also uncommon.

**Table 3:** Dispensed dopaminergic and serotonergic drugs among 1 347 564 older people (≥65 years) according to age groups, 2008. Values are % (n).

Drug type	Total(n = 1 347 564)	Men(n = 572 455)	Women(n = 775 109)	65–69(n = 340 536)	70–74 years(n = 285 461)	75–79 years(n = 259 433)	80–84 years(n = 225 206)	85–89 years(n = 158 672)	≥90 years(n = 78 256)
Dopaminergic drugs	5.6 (75 537)	4.9 (27 782)	6.2 (47 755)	4.2 (14 443)	4.7 (13 345)	5.4 (13 958)	6.4 (14 518)	7.7 (12 266)	9.0 (7 007)
Dopamine enhancing drugs	2.7 (35 911)	2.5 (14 408)	2.8 (21 503)	2.2 (7 445)	2.5 (7 215)	2.9 (7 627)	3.1 (6 995)	3.1 (4 867)	2.3 (1 762)
Dopamine lowering drugs	3.2 (43 129)	2.6 (14 660)	3.7 (28 469)	2.2 (7 654)	2.4 (6 841)	2.7 (7 082)	3.6 (8 219)	5.0 (7 896)	6.9 (5 437)
Serotonergic drugs	13.2 (177 836)	9.3 (53 348)	16.1 (124 488)	9.8 (33 423)	10.1 (28 939)	12.1 (31 481)	15.4 (34 775)	19.7 (31 245)	23.0 (17 973)
Serotonin enhancing drugs	12.4 (167 010)	8.6 (49 442)	15.2 (117 568)	9.4 (31 885)	9.6 (27 535)	11.5 (29 871)	14.5 (32 662)	18.2 (28 915)	20.6 (16 142)
Serotonin lowering drugs	1.6 (21 326)	1.3 (7 242)	1.8 (14 084)	0.8 (2 808)	1.0 (2 772)	1.3 (3 308)	2.0 (4 405)	3.0 (4 736)	4.2 (3 297)

Table 4 shows the results of the logistic regression analysis of whether age and sex were associated with use of dopaminergic and serotonergic drugs. Female gender was related to use of both dopaminergic and, particularly, serotonergic drugs, after adjustment for age. Higher age was associated with use of dopamine lowering drugs and serotonergic drugs, whereas the association with use of dopamine enhancing drugs declined in the oldest old.

**Table 4:** Adjusted odds ratios (ORs) with 95% confidence intervals (95% CIs) for dispensed dopaminergic and serotonergic drugs in 1 347 564 older people, 2008.

	Dopaminergic drugs	Dopamine enhancing drugs	Dopamine lowering drugs	Serotonergic drugs	Serotonin enhancing drugs	Serotonin lowering drugs
	OR (95% CI)	OR (95% CI)	OR (95% CI)	OR (95% CI)	OR (95% CI)	OR (95% CI)
Sex						
Man	Ref	Ref	Ref	Ref	Ref	Ref
Woman	1.22 (1.20–1.24)	1.09 (1.07–1.12)	1.33 (1.31–1.36)	1.75 (1.73–1.77)	1.78 (1.76–1.80)	1.27 (1.23–1.31)
Age (years)						
65–69	Ref	Ref	Ref	Ref	Ref	Ref
70–74	1.10 (1.08–1.13)	1.16 (1.12–1.20)	1.06 (1.03–1.10)	1.03 (1.01–1.05)	1.03 (1.01–1.04)	1.18 (1.12–1.24)
75–79	1.27 (1.24–1.31)	1.35 (1.31–1.40)	1.21 (1.17–1.25)	1.25 (1.23–1.27)	1.24 (1.22–1.26)	1.54 (1.46–1.62)
80–84	1.53 (1.50–1.57)	1.43 (1.38–1.47)	1.61 (1.56–1.67)	1.62 (1.59–1.65)	1.58 (1.56–1.61)	2.36 (2.25–2.47)
85–89	1.85 (1.80–1.90)	1.40 (1.35–1.45)	2.21 (2.14–2.28)	2.13 (2.10–2.17)	2.04 (2.00–2.07)	3.60 (3.44–3.78)
≥90	2.14 (2.07–2.20)	1.01 (0.96–1.07)	3.08 (2.97–3.19)	2.49 (2.44–2.54)	2.27 (2.23–2.32)	5.05 (4.80–5.32)

Table 5 shows combinations of dopaminergic and serotonergic drugs. The occurrence of these combinations was generally low, with dopamine lowering + serotonin lowering drug the most common combination (1.6%). The combinations of dopaminergic and serotonergic drugs were more common in women than in men. However, the relationship with age showed a mixed pattern. The prevalence of combinations of serotonin enhancing + serotonin lowering drug and dopamine lowering + serotonin lowering drug increased with age, whereas the combinations dopamine enhancing + dopamine lowering drug and dopamine enhancing + serotonin enhancing drug showed no clear relationship with age.

**Table 5:** Combinations of dispensed dopaminergic and serotonergic drugs among 1 347 564 older people (≥65 years) according to age groups, 2008. Values are % (n).

Drug combinations	Total(n = 1 347 564)	Women(n = 775 109)	Men(n = 572 455)	65–69 years(n = 340 536)	70–74 years(n = 285 461)	75–79 years(n = 259 433)	80–84 years(n = 225 206)	85–89 years(n = 158 672)	≥90 years(n = 78 256)
Dopamine enhancing + dopamine lowering drug	0.3 (3 503)	0.3 (2 217)	0.2 (1 286)	0.2 (656)	0.2 (711)	0.3 (751)	0.3 (696)	0.3 (497)	0.2 (192)
Serotonin enhancing + serotonin lowering drug	0.8 (10 500)	0.9 (7 164)	0.6 (3 336)	0.4 (1 270)	0.5 (1 368)	0.7 (1 698)	1.0 (2 292)	1.5 (2 406)	1.9 (1 466)
Dopamine enhancing + serotonin enhancing drug	1.3 (17 093)	1.5 (11 451)	1.0 (5 642)	1.2 (4 117	1.2 (3 420)	1.3 (3 404)	1.4 (3 109)	1.4 (2 235)	1.0 (808)
Dopamine lowering + serotonin lowering drug	1.6 (21 326)	1.8 (14 084)	1.3 (7 242)	0.8 (2 808)	1.0 (2 772)	1.3 (3 308)	2.0 (4 405)	3.0 (4 736)	4.2 (3 297)

Table 6 shows the results of the logistic regression analysis of whether age and sex were associated with combinations of dopaminergic and serotonergic drugs. Female gender was associated with all of the combinations of dopaminergic and serotonergic drugs, after adjustment for age. Older age was associated with use of serotonin enhancing + serotonin lowering drug and dopamine lowering + serotonin lowering drug, after adjustment for sex. However, age did not show a clear association with the combinations of dopamine enhancing + dopamine lowering drug and dopamine enhancing + serotonin enhancing drug.

**Table 6:** Adjusted odds ratios (ORs) with 95% confidence intervals (95% CIs) for combinations of dispensed dopaminergic and serotonergic drugs in 1 347 564 older people, 2008.

	Dopamine enhancing + dopamine lowering drug	Serotonin enhancing + serotonin lowering drug	Dopamine enhancing + serotonin enhancing drug	Dopamine lowering + serotonin lowering drug
	OR (95% CI)	OR (95% CI)	OR (95% CI)	OR (95% CI)
Sex				
Man	Ref	Ref	Ref	Ref
Woman	1.25 (1.17–1.34)	1.41 (1.35–1.47)	1.51 (1.46–1.56)	1.27 (1.23–1.31)
Age (years)				
65–69	Ref	Ref	Ref	Ref
70–74	1.29 (1.16–1.43)	1.28 (1.19–1.38)	0.99 (0.94–1.03)	1.18 (1.12–1.24)
75–79	1.49 (1.34–1.66)	1.74 (1.62–1.87)	1.07 (1.02–1.12)	1.54 (1.46–1.62)
80–84	1.58 (1.42–1.76)	2.68 (2.50–2.87)	1.11 (1.06–1.17)	2.36 (2.25–2.47)
85–89	1.59 (1.41–1.78)	3.96 (3.70–4.24)	1.12 (1.06–1.17)	3.60 (3.44–3.78)
≥90	1.22 (1.04–1.43)	4.78 (4.43–5.16)	0.79 (0.73–0.85)	5.05 (4.80–5.32)

## Discussion

### Main findings

In our large nationwide study, dopaminergic drugs were used by 6% and serotonergic drugs by 13% of the elderly population (≥ 65 years). Use of these drugs was associated with female gender and higher age. Drugs that affect the dopaminergic and serotonergic systems are used for a wide range of different medical conditions in older adults. The frequent use of these drugs calls for awareness of cognitive and emotional side-effects in elderly people. This needs to be taken into account when making different clinical evaluations on elderly persons that are based on cognitive or affective deficits.

One important finding in our study is that the use of dopaminergic and serotonergic drugs increases with age. Given what is known about accelerated dopamine losses in the brain from young-old to very-old age [7]–[9], these two opposing trajectories suggest a potential increase of dopaminergic side effect in the older age cohort. This could also be true for serotonergic drugs, even though there are still too few studies on age related decline in the serotonergic system [16], [17].

Previous studies on young women and men have demonstrated a sexual dimorphism in neurotransmission, particularly in the dopamine system [32], [33], related to sex differences in cognitive function [34]. However, given age-related changes in sex hormones levels, the implications of our gender- related findings are difficult to evaluate because of the lack of studies on gender differences in the dopaminergic and serotonergic systems in old age.

Use of dopaminergic and serotonergic drugs can also cause severe somatic side effects in older people. Both dopaminergic and serotonergic drugs have been associated with an increased risk of fractures [35], [36]. In particular, the combination of dopamine enhancing and serotonin enhancing drugs has been associated with increased hip fracture risk [35]. This drug combination was related to female gender in our study, which may be problematic as women are at greatest risk of fractures [37]. Further, advanced age and use of serotonergic drugs have previously been associated with an increased risk of bleeding [31]. In our study, advanced age was correlated to use of these drugs.

However, dopaminergic and serotonergic drug therapy may also be efficacious for treatment of depression, psychotic disorders and Parkinson's disease in old age [38]–[41]. Nonetheless, older people are often underrepresented in clinical trials [42] and, therefore, there is a lack of high-level evidence for treatment with dopaminergic and serotonergic drugs in this population.

### Limitations

We analysed data on 1 347 564 elderly people registered in the Swedish Prescribed Drug Register from July to September 2008, which corresponded to about 81% of the total population aged ≥65 years in Sweden (according to Statistics Sweden's census data from 30 September 2008). Hence, about 19% of the of the total Swedish population aged ≥65 years did not fill a prescription at a Swedish pharmacy during the study period. The generalizability of our results to other similar countries is postulated to be high given that we have analyzed an unselected population. The Swedish Prescribed Drug Register does not include information about diagnoses or over-the-counter drugs. In 2008, some antimigraine selective serotonin (5HT1) agonists were available over the counter in Sweden, which may lead to an underestimation of the use of these drugs in our study. Also, the register does not include drugs used in hospitals or from drug storerooms, which may lead to an underestimation of drug use in the institutional setting.

Moreover, our method is built on an assumption that all current drugs were dispensed during the observed 3 month period, due to the fact that drugs are prescribed for use during at most 90 days in Sweden. Therefore, we might have disregarded drugs that were dispensed before the three month period and used at a slower rate than intended. At the same time, we might have included drugs that were dispensed during the three month period but discontinued prematurely. In addition, our method is based on interpretations of the dispensed drugs' dosages written by the prescribers, as well as assumptions about DDDs when information about dosage was incomplete or missing [26], [27]. Finally, a general limitation of studies on drug registers is that data may not adequately reflect actual drug use given that adherence to treatment can be low [43].

### Conclusion

Approximately one out of ten older patients uses serotonergic drugs and one out of twenty dopaminergic drugs. The frequent use of dopaminergic and serotonergic drugs in the elderly patients is a potential problem due to the fact that aging is associated with a down-regulation of both these monoaminergic systems. Future studies are needed for evaluation of the impact of these drugs on different cognitive and emotional functions in old age.
